# MicroRNA-34b mediates hippocampal astrocyte apoptosis in a rat model of recurrent seizures

**DOI:** 10.1186/s12868-016-0291-6

**Published:** 2016-08-11

**Authors:** Liqun Liu, Lingjuan Liu, Jiayun Shi, Menglin Tan, Jie Xiong, Xingfang Li, Qingpeng Hu, Zhuwen Yi, Ding’an Mao

**Affiliations:** Department of Pediatrics, The Second Xiangya Hospital of Central South University, 86 Renmin Middle Rd, Changsha, 410011 Hunan People’s Republic of China

**Keywords:** MiR-34, Bcl-2, Convulsion, Hippocampus, Astrocytes

## Abstract

**Background:**

Recurrent convulsions can cause irreversible astrocyte death, impede neuron regeneration, and further aggravate brain damage. MicroRNAs have been revealed as players in the progression of numerous diseases including cancer and Alzheimer’s disease. Particularly, microRNA has been found linked to seizure-induced neuronal death. In this study, a rat model of recurrent convulsions induced by flurothyl treatments was utilised to assess the alterations of microRNA expressions in hippocampus tissues. We also applied an in vitro model in which primary astrocytes were exposed to kainic acid to verify the targets of miR-34b-5p identified in the animal model.

**Results:**

We discovered that miR-34b-5p, a member of the miR-34 family, increased significantly in flurothyl-treated rat hippocampus tissue. More surprisingly, this upregulation occurred concurrently with accumulating astrocyte apoptosis, indicating the involvement of miR-34b-5p in seizures caused astrocyte apoptosis. Results from the in vitro experiments further demonstrated that miR-34b-5p directly targeted Bcl-2 mRNA, translationally repressed Bcl-2 protein, and thus modulated cell apoptosis by influencing Bcl-2, Bax, and Caspase-3.

**Conclusion:**

Our findings prove microRNAs play a role in mediating recurrent convulsions-induced astrocyte death and further indicate that miR-34b-5p could acts as a regulator for astrocyte apoptosis induced by recurrent seizures.

**Electronic supplementary material:**

The online version of this article (doi:10.1186/s12868-016-0291-6) contains supplementary material, which is available to authorized users.

## Background

Status epilepticus (SE), a prolonged seizure, has a higher prevalence in children than adults. Nearly 40 % of SE occurs in infants less than 2 years [1]. Recurrent convulsions associated with SE during neonatal development cause irreversible brain damage. Children develop impairments in physical activity, cognition, and language abilities, devastating both patients and caregivers [2–6]. Exploring the mechanisms and treatments of recurrent convulsions has become an emerging primary focus in clinical research.

Astrocytes play a critical role in enabling the central nervous system (CNS) to maintain its normal structure and function [6–8]. Disrupted astrocyte function is linked to various CNS diseases and the relation of astrocytes with SE has been unveiling. There is emerging evidence showing that astrocytes involve in the pathological remodeling process of SE. Their dysfunctions have been linked to the failure of brain homeostasis in epileptic patients who suffered from hippocampal sclerosis as well as kainic acid-induced mouse SE model [9–11]. MicroRNA, an evolutionally conservative class of small non-coding RNA, extensively affects the progression of diseases including neurodegenerative disease, traumatic brain injury, and brain cancer [12]. Specifically, a series of data demonstrate that microRNAs can regulate neural cell specification. Several microRNA families have also been shown to be involved in regulating the proliferation and survival of astrocytes, suggesting that microRNAs play essential roles in astrocyte functions [13–15].

The miR-34 family is composed of three mature non-coding microRNAs-miR-34a, miR-34b, and miR-34c-found at three different loci across the genome. Among these, miR-34a has been studied extensively and considered a promising target for cancer therapies [16]. Previous studies have revealed that miR-34a interplays with key cell proliferation and death molecules in cancer cells, including p53, the Bcl-2 family, c-Myc, and PDGF. Specifically, p53-activated miR-34a inhibits cell growth by downregulating the expression of E2F, c-Myc, CDK4, CDK6, CyclinD1, and CyclinD2 [17–19]. Overexpression of miR-34a in several cancer cell lines induces pro-caspase-3 activation and PARP disassociation, resulting in caspase-3-dependent cell death [20]. Moreover, miR-34a mRNA level is negatively correlated with Bcl-2 protein level and directly modulates Bcl-2 expression in hepatocellular carcinoma (HCC) [21]. In the CNS, Agostini et al. [22] show that miR-34a can mediate SYT-1 and Syntaxin-1A expression, inhibit their functions, and reshape hippocampal spinal morphology, suggesting potential roles for miR-34s in CNS diseases. Sano et al. [23] also demonstrated the upregulation of micro-34a during seizure-induced neuronal death, further indicating the involvement of miR-34 family in seizure pathology.

Currently, the majority of research in this field emphasises the study of miR-34a. But the function and regulation of miR-34b and miR-34c remain poorly understood, especially regarding their roles in the CNS. In this paper, we discovered that one subtype of miR-34b—miR-34b-5p—mediated astrocyte apoptosis caused by recurrent flurothyl-induced seizures in the way in which they directly targeted Bcl-2.

## Methods

### Animals and animal experimental designs

The rat model of recurrent convulsions was established by exposing rats to flurothyl. A total of 96 20-day-old Sprague–Dawley rats were randomly divided into control and flurothyl-treated groups. To expose a rat to flurothyl, the rat was relocated to a chamber (40 cm × 20 cm × 20 cm), and the relocation was followed by an infusion of 0.1 ml flurothyl (bis(2,2,2-trifluoroethyl), Sigma-Aldrich, USA) to a gauze pad suspended inside the chamber. Once the flurothyl infusion step was complete, the chamber was sealed by the experimenters. Because flurothyl is a highly volatile chemoconvulsant, the rats assigned to the flurothyl-treated group were exposed to the chemical immediately. Thirty minutes after the first generalised seizure appeared in a rat, the rat was removed from the chamber to a normal environment. This flurothyl-seizure induction protocol was performed once a day for six consecutive days. The rats in the control group underwent the same operations including the relocation to the chamber and exposure to solutions. But the rats in the control group did not be exposed to flurothyl. To collect samples at several different time points according to our experimental design, we anaesthetised rats with ketamine (40 mg/kg) and lidocaine (0.4 % 10 mg/kg), then performed cardio-perfusion with 40 ml PBS followed by brain tissue extraction. Time points included in this experiment were: 24 h before flurothyl treatment, 2 h after flurothyl treatment, 6 h after flurothyl treatment, 1 day after flurothyl treatment, 3 days after flurothyl treatment and 7 days after flurothyl treatment. Each time point further included control group and seizured group (n = 8/group/time point). All animals used in this project were provided by the College of Animal Technology, Hunan Agricultural University. Rats were kept on a 12-h light–dark cycle with lights turned on at 6:00 a.m. and were given unlimited access to food and water.

### MicroRNA isolation, RNA isolation, reverse transcription, and quantitative real time PCR

Using an Ambion^®^ mirVana™ MicroRNA Isolation Kit (TaqMan), we extracted total microRNAs from the rat hippocampus tissues at different time points after the 6-day flurothyl treatments were completed. Total RNAs from hippocampus tissues and cultured astrocytes were isolated using TRIzol (Invitrogen, USA). Reverse transcription of microRNAs was performed using the TaqMan MicroRNA Reverse Transcription system. Quantitative real time PCR (qPCR) was carried out with TaqMan Universal PCR Master Mix and TaqMan rodent microRNA fluidic cards. The amplification process was done with a 7900 HT fast real time PCR system (Applied Biosystems). Data were analysed by SDS2.4 (ABI). Reverse transcription of mRNA was performed using a Fermentas reverse transcriptase system. Quantitative real time PCR for the rat miR-34b-5p (RiboBio, 5201) and U6 snRNA (RiboBio, 0201) was measured using a Thermo Scientific Maxima SYBR Green/ROX qPCR Master Mix (2×) (Cat. K0221) following the procedures laid out by the manufacturer. The primers used in qRT-PCR are listed as follows: Bcl-2 F: 5′-GGCATCTTCTCCTTC CAG-3′, R:5′-CATCCCAGCCTCCGTTAT-3′; β-actin:F:5′-GAGGGCATGGGTCAGAAG-3′,R:5′-GAGGCGTACAGGGATAGCAC-3′.

### MicroRNA transcriptome

The reverse transcription step started by mixing the following reagents: 0.8 μl Megaplex™ RT Primers (10×), 0.2 μl dNTPs with dTTP (100 mM), 1.5 μl MultiScribe™ Reverse Transcriptase (50 U/μl), 0.8 μl 10× RT Buffer, 0.9 μl MgCl_2_ (25 mM), 0.1 μl RNase Inhibitor (20 U/μl), 0.2 μl Nuclease-free water, and 3 μl total extracted RNA. The mixture was then kept on ice for 5 min before put in Real-Time PCR System(7900HT) (Applied Biosystem, USA) for reaction. The amplification steps are described here: 40 cycles of 16 °C 2 min, 42 °C 1 min and 50 °C 1 s, followed by 5 min 85 °C and 4 °C forever. The PCR products was store in −20 °C for use.

To perform microarray experiments, we used 900 μl PCR mix for each miRNA TLDA (Taqman Low density array, Applied Biosystem). The PCR mix was composed of 450 μl TaqMan^®^ Universal PCR Master Mix, 6 μl RT product, and 444 μl Nuclease-free water. Later, this PCR mix was added to TaqMan Rodent MicroRNA A + B cards and placed in 7900HT real-time PCR machine for the reaction. The reaction condition was: 95 °C 10 min; 40 cycles of 15 s 95 °C, 60 s 60 °C. Each sample had quadruplicate. U6 was used as loading control.

The results were analysed using SDS2.4 software (ABI).

### Cell culture

McCarthy methods for primary rat hippocampal astrocyte culture have been described previously [24]. 2-day Newborn Sprague–Dawley rats were euthanised with CO_2_ and sterilised thoroughly by immersion in 75 % ethanol for 5 min. Under sterile conditions, the rat brains were collected and washed twice with cold D-Hanks solution. Under a microscope, we carefully detached the meninges and adjoining blood vessels from brain tissues and then separated the hippocampus from the whole brain. We then cut the hippocampus into small pieces and transferred the chopped hippocampus tissue to a 15 ml conical tube containing 1 ml 0.25 % trypsin (Sigma, St Louis, MO, USA) for digestion. The digestion step was completed below 37 °C for 20 min and stopped by adding a culture medium containing fetal bovine serum. Following the digestion step, we filtered the digested solution through 200-mesh CellCribble, collected filtrate, and centrifuged the filtrate for 10 min at 1000 rpm. We then discarded the supernatant, resuspended the cells with the astrocyte culture medium, and moved the suspended solution to a 25 cm^2^ flask. Then, we enriched the astrocyte concentration by a differential adhesion method in which a 60-min centrifugation was used to separate astrocytes from attached mesenchymal cells. The astrocyte culture medium, we used was DMEM based, supplemented with 20 % fetal bovine serum (Hyclone, USA), 2 mmol/l l-glutamine, and penicillin–streptomycin (100 μmol/ml). For this experiment, 1 × 10^6^/well enriched astrocyte cells were seeded on a 25 cm^2^ flask coated with poly-l-lysine and supplied with 5 % CO_2_ at 37 °C.

### Western blot

Protein samples from the hippocampus tissues and primary astrocytes were loaded to a 12 % SDS-PAGE. PVDF membranes were used for the SDS-PAGE. The membrane was blotted for 2 h using 5 % non-fat milk in the TBST solution. Antibodies against Bcl-2 (1:200), Caspase-3 (1:1000), and Actin (1:1000) were purchased from Santa Cruz Biotechnology. HRP conjugated secondary antibodies against mice IgG and rabbit IgG (1:5000) were purchased from Proteintech Group, USA. Quantitative data of western blots were analysed using Gel-Pro4.0 software. The intensity of the tested protein bands was normalised to the internal reference.

### TUNEL

In situ terminal deoxynucleotidyltransferase mediated dUTP nick end labeling of fragmented DNA (TUNEL) was used to analyse the apoptosis of hippocampal neurons and primary cultured astrocytes. Rat brain tissues were collected 1 day after the 6-day treatment was completed and then embedded with paraffin. Hippocampal neuron apoptosis was observed by staining brain tissues with an in situ cell death TUNEL kit (MK1020, Boster, China). Primary rat hippocampal astrocytes were seeded in a six-well plate at a density of 1 × 10^5^/well. After reaching 90 % of confluence, astrocytes were treated with 50 μM, 100 μM, 150 μM, or 200 μM kainic acid for 24 h, followed by TUNEL staining (Roche, USA). Fluorescent microscopy was then used to observe the results (Nikon, USA). We randomly chose five views under the microscope for each treatment group and calculated the number of TUNEL positive cells and total cells. The apoptotic rate was calculated by dividing the number of TUNEL positive astrocytes by the total number of cells. All images were analysed with NIS-Elements BR 3.0 provided by Nikon.

### Transfections of plasmids and MicroRNA mimics and inhibitors

Transfections of plasmids and microRNA mimics and inhibitors were conducted using Lipofectamine2000 (Invitrogen), following the procedures laid out by the manufacturer. Cells were plated in six-well plates. MiR-34b-5p mimics (100 nM, Ribobio, Guangzhou, China); miR-34b-5p inhibitors (100 nM, Ribobio, Guangzhou, China); Bcl-2 siRNA (50 nM) (Qiagen, Hilden, Germany); and controls including miR-34b-5p m NC, miR-34b-5p i NC, and control siRNA were transfected into primary astrocytes when they reached 60–70 % confluence. In a luciferase assay, WT-pGL3-Bcl-2-3′ UTR, MUT-pGL3-Bcl-2-3′ UTR, or pGL3-control plasmid (200 ng, Ribobio, Guangzhou, China) was co-transfected with control plasmid phRL-nμll (80 ng) into hippocampal astrocytes cultured in 24-well plates. MiR-34b-5p mimics and inhibitors as well as miR-34b-5p i NC and miR-34b-5p m NC were designed by Ribobio, Guangzhou, China. Available sequences are as follows: Bcl-2 siRNA sense 5′-UGGAUGAUCGAGUACCUGAdT dT-3′; antisense5′-UCAGGUACUCAGUCAUCCAdCdA-3′; control siRNA sense: 5′-UUCUCGAACGUGUCAC GUdTdT-3′; antisense:5′-ACGUGACACGUUCGUUCGGCGAAdTdT-3′.

### Luciferase reporter assay

Cells at density of 1 × 10^5^/well were plated in 24-well plates 24 h before transfection. Luciferase constructs containing firefly luciferase with either WT Bcl-2 3′-UTR or mutant Bcl-2 3′-UTR were co-transfected into the cells with luciferase construct containing Renilla luciferase driven by thymidine kinase promoter (an internal control). Cell lysates were collected 48 h after the transfection, and the luciferase activity measurement was done following the procedures laid out by the manufacturer (Dual-Luciferase system, Promega, E1910). WT Bcl-2 plasmid was cloned using F:5′-GAATCTAGAGGTCGACAAACCTGCCCCAAAC-3′ R:5′-AATGGCCGG CCTGGCAGTAAATAGCTGATTCGAC-3′. Mutant Bcl-2 was cloned using the following primers: (underlining indicates mutation sites) F:5′-GAATCTAGAGGTCGACAAACCTGCCCCAAAC-3′ R: 5′-AATGGCCGGCCTCCCACTAA ATAGCTGATTCGACC-3′.

### Statistical analysis

For the purpose of miRNA profiling, a twofold change with *P* < 0.05 was considered significant. Data in this study are presented as mean SEM and were analysed by SPSS 18.0. Data was analysed with Student’s *t* test or one-way ANOVA followed by an LSD-*t* test. *P* < 0.05 was considered statistically significant. N of each statistical analyses is included in figure legends.

## Results

### Induced apoptosis in rat hippocampus after recurrent seizures

**Fig. 1:**
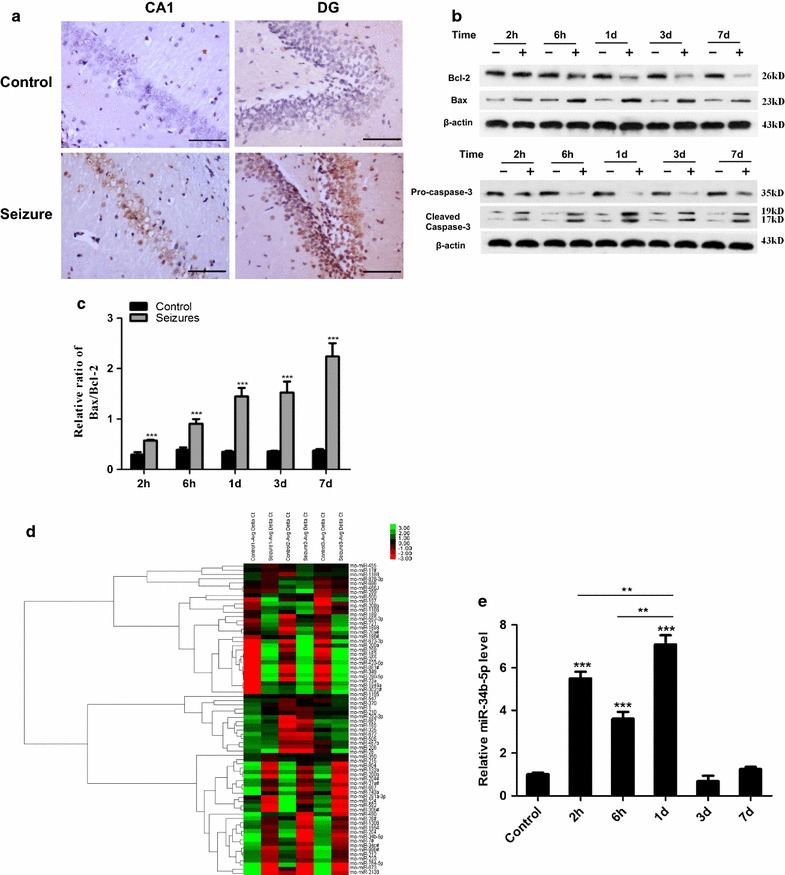
Cell apoptosis and miR-34b-5p expression induced in rat hippocampus after flurothyl treatments. **a** Pictures of TUNEL staining (400) showed seizured mice with more cell death in both the CA1 and DG regions. DAB detection; representative of eight mice; scale bars, 100 μm. **b** Western blotting. Hippocampal tissue protein was extracted from control and flurothyl-treated rats at 2 h, 6 h, 1 day, 3 days, or 7 days after treatments were completed. Bcl-2, Bax, pro-caspase-3, and caspase-3 were blotted for analyses. **c** Bax/Bcl-2 ratio (*n* = 8, comparing seizured rat tissue with control rat tissue at different time points, 2 h: *t*_14_ = 8.53; 6 h: *t*_14_ = 8.33; day 1: *t*_14_ = 12.83; day 3: *t*_14_ = 10.94; day 7: *t*_14_ = 14.72; ***P < 0.0001). **d** Heat map summarising the patterns of expression for 72 microRNAs that were differentially expressed in hippocampal tissue of either the control or the flurothyl-treated group at one day after treatments were completed (n = 3). **e** Relative level of miR-34b-5p. n = 8 animals in each group (one-way ANOVA, *n* = 8, *F*_5,42_ = 157.5; **1 day versus 6 h or 2 h, *P* < 0.01; ***1 day, 6 h or 2 h versus control *P* < 0.0001)

Neural cell apoptosis in the hippocampus had been observed in tissue samples from epileptic patients since the early nineteenth century. Staining and western blotting of samples from human patients with temporal lobe epilepsy showed evidence of altered apoptotic signaling [25, 26]. Specifically, in both rodent models and human biopsy samples, astrocyte apoptosis was observed and considered to serve as a primary event happening during post-convulsion pathological development [27]. In order to study the role of microRNAs in mediating brain damage related to recurrent convulsions, we used a rat model of recurrent convulsions in which we exposed rats to flurothyl for six consecutive days. All the rats assigned to the flurothyl treatment group displayed a phenotype of recurrent convulsions, while those in the control group remained normal. We also analysed cell death in hippocampus tissue using TUNEL staining. In conformity with previous studies, we observed a higher percentage of TUNEL-positive cells in the hippocampus tissues from rats in the flurothyl-treated group than we did in tissues from the control group (Fig. 1a).

Bcl-2 protein has been thought of as an anti-apoptotic protein, while protein Bax, in the same family, has been considered a pro-apoptotic protein. The ratio of Bax to Bcl-2 levels was used as a determining checkpoint for cell apoptosis [28]. Autopsy studies in epileptic patients showed altered protein levels of Bcl-2 and Bcl-xl families in relation to neuron apoptosis, indicating that apoptotic events occurred in post-seizure periods [29]. To determine how the Bax/Bcl-2 ratio was altered in our rat model of recurrent seizures in relation to cell apoptosis, we collected hippocampal tissue samples from rats in the flurothyl-treated group and the control group at five time points: 2 h, 6 h, 1 day, 3 days, and 7 days after all the flurothyl treatments had been completed. We then evaluated the Bax, Bcl-2, and caspase-3 protein levels of all collected samples. For all time points we examined, the Bcl-2 protein level slowly decreased over time, along with a gradual increase of Bax protein. The protein level of Bcl-2 did not show a significant difference between the control and flurothyl-treated group at 2 h after the 6-day treatment was completed (*t*_14_ = 0.81, *P* = 0.432). However, starting at 6 h after the treatments were finished, a difference in Bcl-2 protein levels began to appear between the control group and the flurothyl-treated group. By day 1, day 3, and day 7 after treatment completion, the Bcl-2 protein level of rat hippocampus in the flurothyl-treated group was statistically significantly lower than the level in the control group (day 1:t_14_ = 15.46; day 3: *t*_14_ = 17.83; day 7: *t*_14_ = 28.29; for all time points: *P* < 0.0001). Conversely, starting at 2 h after treatments were finished, the Bax protein level of rat hippocampus in the flurothyl-treated group gradually increased while remaining stable in the control group (Fig. 1b). And, Bax protein levels do present significantly increase starting from 2 h from treatment ended to 7 days after flurothyl treatment ended (2 h:*t*_14_ = 13.10; 6 h: *t*_14_ = 10.01; day 1:*t*_14_ = 19.24; day 3:*t*_14_ = 9.04; day 7: *t*_14_ = 7.4; for all time points: *P* < 0.0001). Overall, the Bax/Bcl-2 ratios of rat hippocampus in the flurothyl-treated group at all time points were statistically significant higher than the ratios for the control group (2 h: *t*_14_ = 8.53; 6 h: *t*_14_ = 8.33; day 1:*t*_14_ = 12.83; day 3:*t*_14_ = 10.94; day 7: *t*_14_ = 14.72; for all time points: *P* < 0.0001). Moreover, the Bax/Bcl-2 ratio continued to increase through all time points during the post-seizure period, while remaining low and stable in the control group (Fig. 1c). Similarly, the cleaved caspased-3 level of rat hippocampus in the flurothyl-treated group stayed significantly higher than the level in the control group for all post-seizure time points we examined (*P* < 0.0001 for all time points). The highest level of cleaved caspase-3 appeared at 1 day after the flurothyl treatments were completed, indicating that the prevalence of apoptotic cells peaked at this time point (Fig. 1b). These data demonstrated that cell death occurred in the hippocampus tissues of flurothyl treated rats as a result of seizure.

### MicroRNA transcriptome in rats treated with flurothyl and the induction of miR-34b-5p

Given the existence of neuron apoptosis in several CNS diseases and the accumulated evidence showing that microRNAs mediate cell apoptosis in the CNS, we decided to explore microRNA transcriptome using an array-based microRNA profiling technique [30, 31].

We characterised and compared the microRNA profiles of the hippocampus tissues from experimental and control groups (Fig. 1d). In total, we selected microRNAs changes more than twofold with p value smaller than 0.05 and listed the top 30 altered microRNAs whose expressions were either upregulated or downregulated the most. Among these altered microRNAs, nine microRNAs were upregulated and 21 microRNAs were downregulated (Table 1). Among these candidates, miR-34b-5p attracted our attention because of previous studies about the miR-34 family in CNS diseases. Compared to miR-34b-5p, several other microRNAs, such as miR-1306, changed more dramatically. However, these microRNAs had either already been studied in detail by other groups or else shown insufficient evidence to date of association with diseases. MiR-34b, miR-34c, which locate in the same transcriptional unit as miR-34b, were moderately upregulated. Our array data showed that the miR-34b-5p level was upregulated seven fold in the hippocampus tissues of rats treated with flurothyl. To re-confirm this upregulation of miR-34b-5p discovered in the array data, we performed quantitative stem-loop polymerase chain reaction (qRT–PCR) in the hippocampus tissue samples collected at different time points and successfully validated the significant upregulation of miR-34b-5p in hippocampus tissues from rats treated with flurothyl (Fig. 1e, *P* < 0.0001). By 2 h after the 6-day flurothyl treatment, a fivefold increase of miR-34b-5p was observed. By day 1 when most pro-caspase-3 was cleaved into active caspase-3, a peak sevenfold induction in miR-34b-5p level was observed. By day 3 and day 7, the expression level of miR-34b-5p gradually decreased to normal. One observation, that the trend of miR-34b-5p level was similar to the trend of cleaved caspase-3 across all post-seizure time points we studied, indicated that the initiation of cell apoptosis events could be correlated with the changes of miR-34b-5p level (Fig. 1a, e).

**Table 1:** miRNAs alterations in rat hippocampus after recurrent seizures

miRNA	Fold changes	Regulation	miRNA	Fold change	Regulation
miR-1306	21.7655	Up	miR-500	6.8923	Down
miR-448	21.0113	Up	miR-215	6.0471	Down
miR-224	16.6746	Up	miR-299	4.6631	Down
miR-204#	9.98247	Up	miR-197	4.3048	Down
miR-34b-5p	7.04904	Up	miR-17#	4.1252	Down
miR-34c#	5.93634	Up	miR-1	4.0987	Down
miR-28#	5.13192	Up	miR-505	4.0758	Down
miR-204	4.92734	Up	miR-350	3.4316	Down
miR-592	2.32224	Up	miR-28	3.3122	Down
miR-183	257.0694	Down	miR-98	3.2761	Down
miR-696	81.6994	Down	miR-450a-5p	3.2400	Down
miR-582-3p	24.2659	Down	miR-29b	3.2111	Down
miR-721	11.3161	Down	miR-203	3.1991	Down
miR-672	7.5489	Down	miR-1188	3.0174	Down
miR-493-3p	7.4394	Down	miR-206	2.6099	Down

### MiR-34b-5p mediates kainic acid-induced astrocyte apoptosis

To distinguish which hippocampus cell populations were undergoing apoptosis, we carefully analysed hippocampal TUNEL staining results and found that a certain percentage of TUNEL positive cells was residing in the hilus and subgrandule zone of the hippocampal dentate gyrus, where abundant astrocytes are supposed to reside [32]. Given the fact that the upregulation of miR-34b-5p was observed and the previous study showing an association between astrocyte apoptosis and convulsions, we hypothesised that miR-34b-5p functionally contributed to astrocyte apoptosis in recurrent convulsions [27].

**Fig. 2:**
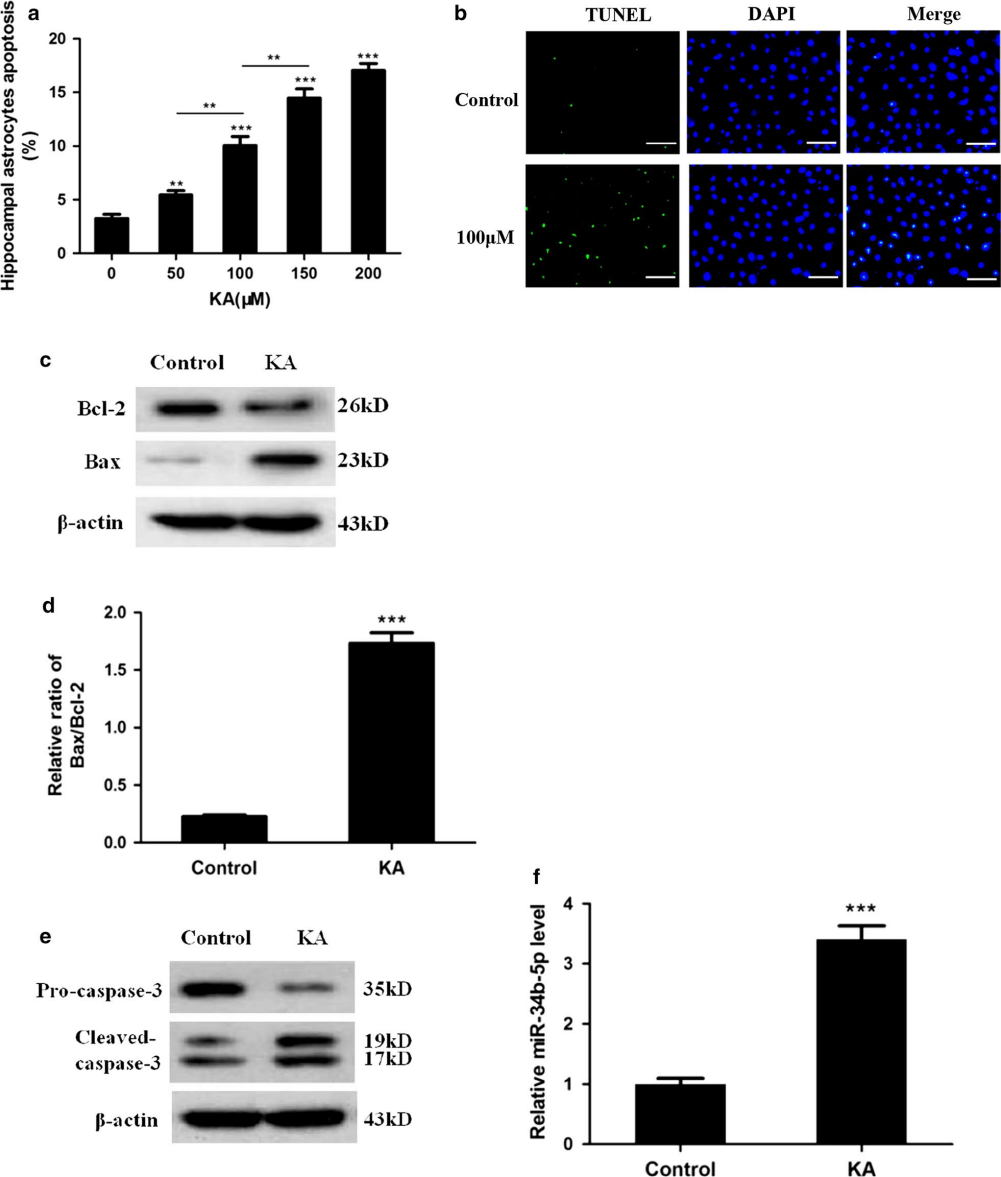
MiR-34b-5p upregulation in kainic acid induced astrocyte apoptosis. **a** Primary astrocytes were treated with 50 μM, 100 μM, 150 μM or 200 μM kainic acid for 24 h followed by TUNEL staining and quantification for the percentage of TUNEL positive cells. *n* = 5 for each treatment group (one-way ANOVA test, *F*_4,20_ = 70.24, ***P < 0.0001; **50 μM KA versus control, 50 μM KA versus 100 μM and 100 μM versus 150 μM *P* < 0.01, *** individual KA treatment versus control *P* < 0.0001). **b** Representative images of three repeated TUNEL staining experiments after 24-h kainic acid treatment; TUNEL (green), DAPI (blue); scale bar 50 μm. **c**–**e** Western blot and quantification. Cell lysate of 24 h 100 μM KA treated primary astrocytes was analysed with SDS-PAGE. Bcl-2, Bax, Pro-caspase-3, and caspase-3 were blotted. The significance difference of Bax/Bcl-2 was observed (*n* = 5, *t*_8_ = 12.93, ***P < 0.0001). **f** Quantitative PCR result: miR-34b-5p upregulated after 100 μM kainic acid treatment (*n* = 5, *t*_8_ = 9.775, ***P < 0.0001)

**Fig. 3:**
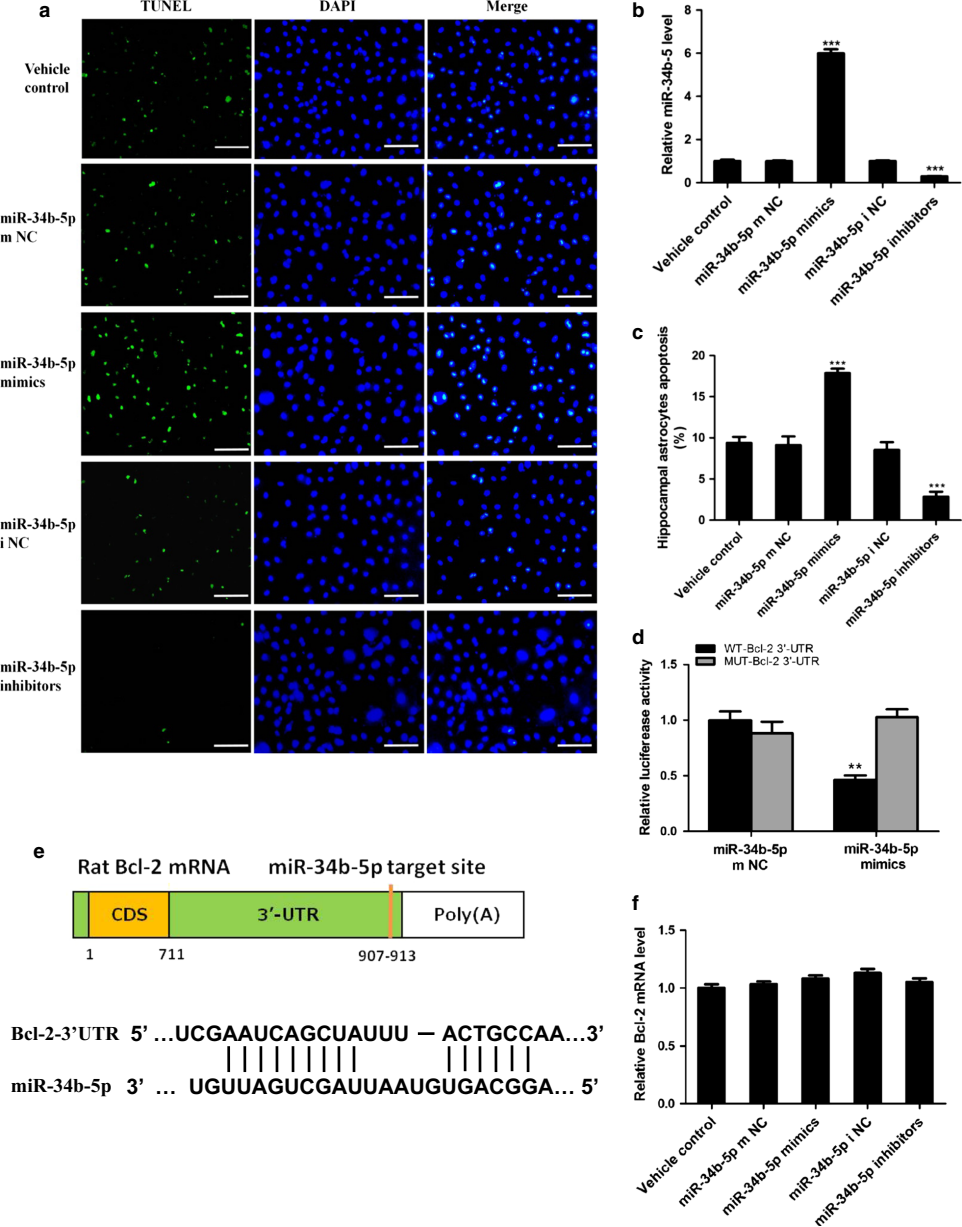
miR-34b-5p promotes KA-induced astrocytes apoptosis by targeting Bcl-2. **a** Representative images of three repeated TUNEL staining experiments on cultured astrocytes. The astrocytes are pre-treated with of miR-34b-5p mimics, inhibitors, mimics negative control (mNC), and inhibitors negative control (i NC), followed by KA treatment; *scale bar* 50 μm. **b** Relative miR-34b-5p levels after different treatments; (one-way ANOVA, *n* = 5, *F*_4*,20*_ = 866.9, ***P < 0.0001). **c** Percentage of TUNEL positive astrocytes over total counted astrocytes represented as apoptotic rate. Cells were treated with the four indicated treatments before fixing and performing TUNEL staining experiments (one-way ANOVA, *n* = 5, *F*_4,20_ = 67.45, ***P < 0.0001). **d** Luciferase assay. Astrocytes was transfected with WT Bcl-2 3′-UTR or Mutant Bcl2 3′-UTR followed by treating either with control or miR-34b-5p mimics. Luciferase activity is indicated by the ratio of firefly luciferase activity to Renilla luciferase activity (Between miR-34b-5p mimics treated with WT-UTR or MUT-UTR, n = 3, *t*_4_ = 7.10, **P = 0.0021). **e** Graphic picture shows miR-34b-5p sequence match with rat Bcl-2 mRNA 3′-UTR. **f** Quantitative PCR shows Bcl-2 mRNA level in response to different treatments (one-way ANOVA, *n* = 5, *F*_4,20_ = 0.4576, *P* = 0.7658)

In the 1970s, kainic acid (KA) was found to induce hippocampal neuronal apoptosis, and it has been widely used as an experimental drug for convulsion models since then [33, 34]. Specifically, KA has been shown to activate glutamate receptors, induce ROS, affect mitochondria function, and cause cell death in astrocytes [35]. Here, we tested whether miR-34b-5p mediated KA induced astrocyte apoptosis in vitro. We exposed primary astrocytes to different concentrations of KA and found that the apoptotic rate increased significantly as the concentration of KA rose (*P* < 0.0001, Fig. 2a and Additional file 1: Fig. 1). A 100 μM KA treatment sufficiently induced about 10 % cell apoptosis detected by TUNEL staining (Fig. 2a, b). Also, astrocytes treated with 100 μM KA expressed a high level of cleaved caspase-3, Bax and Bax/Bcl-2 ratio but low levels of Bcl-2 and pro-caspase-3 (Fig. 2c–e). More importantly, in accord with our results from the in vivo rat model, we detected increased miR-34b-5p expression in KA treated astrocytes (*t*_8_ = 9.775, *P* < 0.0001; Fig. 2f). To further confirm the correlation between miR-34b-5p level and KA-induced astrocyte apoptosis, we manipulated miR-34b-5p levels by treating primary astrocytes either with miR-34b-5p mimics or with miR-34b-5p inhibitors, followed by KA treatment. MiR-34b-5p mimics efficiently increased miR-34b-5p levels six-fold (difference among groups: *F*_4,20_ = 866.9, *P* < 0.0001; Fig. 3b). Nearly 17 % of the total astrocytes underwent apoptosis in the group treated with mimics, while only about 10 % of the total astrocytes in the control group underwent apoptosis (Fig. 3a, c). In contrast, pre-treating primary hippocampal astrocytes to miR-34b-5p inhibitors before KA treatment caused significant reduction in miR-34b-5p levels, and the percentage of apoptotic cells was greatly lowered, to less than 5 % (difference among groups: *F*_4,20_ = 67.45, *P* < 0.0001, Fig. 3a, c). These two results strongly support our hypothesis that miR-34b-5p mediates astrocyte apoptosis in response to KA treatment in vitro.

### MiR-34b-5p mediates cell apoptosis by targeting Bcl-2

**Fig. 4:**
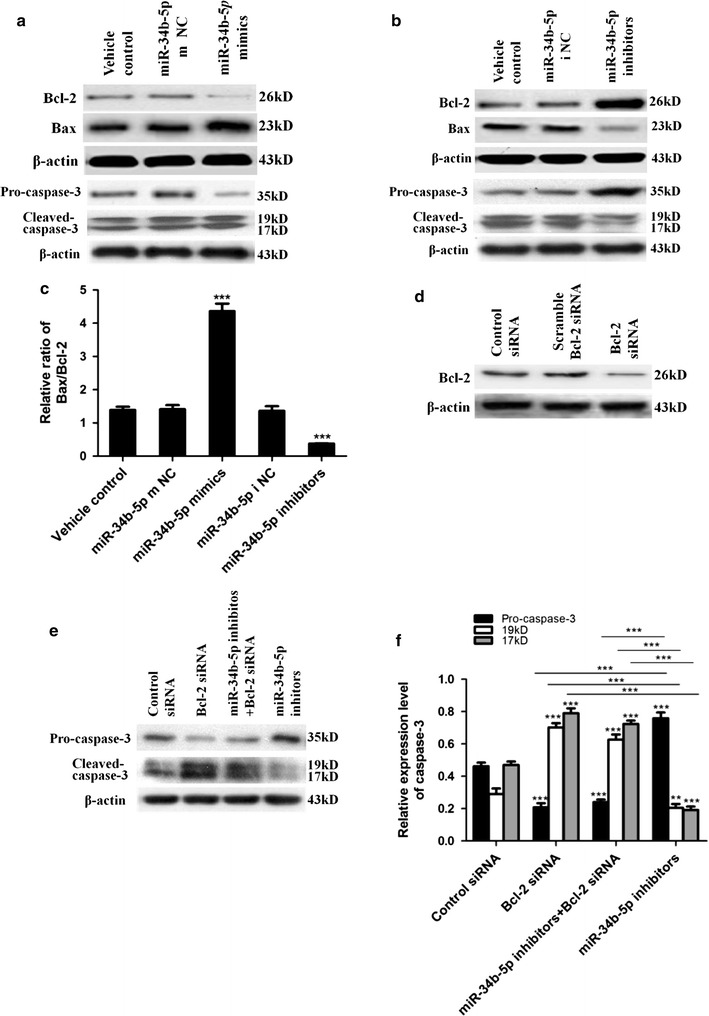
miR-34b-5p regulation of astrocyte apoptosis through mediating Bcl-2 expression level. **a**, **b** Western blot analysis for Bcl-2, Bax, pro-caspase-3, and cleaved caspase-3. Cell lysates were prepared from astrocytes treated with vehicle control, mimics negative control (m NC) or inhibitors negative control (i NC), miR-34b-5p mimics or inhibitors. **c** Bax/Bcl-2 protein ratio after quantitative analysis (one-way ANOVA, *n* = 5, *F*_4,20_ = 123.8, ***P < 0.0001). **d** Western blot protein analysis for Bcl-2 and actin. Cell lysates were prepared from cultured astrocytes treated with control siRNA, scramble siRNA, and Bcl-2 siRNA. **e** Western blot analysis for pro-caspase-3, cleaved caspase-3, and actin. Cell lysates were prepared after astrocytes were treated with control siRNA, Bcl-2 siRNA, miR-34b-5p inhibitors and Bcl-2 siRNA together, and miR-34b-5p inhibitors alone. **f** Quantitative analysis for results in E (one-way ANOVA, pro-caspase-3 group, *n* = 5, *F*_3,16_ = 211.6, ***P < 0.0001; Caspase-3-19KD group, *n* = 5, *F*_3,16_ = 98.36, ***P < 0.0001; Caspase-3-17 KD group, *F*_3,16_ = 158.7, ***P < 0.0001; ***on top of bars, comparing with control within same group; ***on top of lines, comparing between the individual *bars the line covers*, **P < 0.01, ***P < 0.0001)

Based on the results above, we further asked the question of how miR-34b-5p mediated astrocyte apoptosis. Using starBase (http://starbase.sysu.edu.cn/), we screened potential targets for miR-34b-5p based on sequence targeting analysis. Among the hits we identified, Bcl-2 was a potential target for miR-34b-5p (Fig. 3e). With the aim of validating whether Bcl-2 was targeted by miR-34b-5p, we transfected astrocytes with luciferase reporter constructs containing either wild type Bcl-2 3′-UTR or mutant Bcl-2 3′UTR. Next, we treated those transfected astrocytes with either miR-34b-5p mimics or control mimics. The miR-34b-5p level was successfully increased upon mimics transfection (Additional file 2: Fig. 2A). The luciferase activity of WT Bcl-2 3′-UTR was significantly lowered by treatment with miR-34b-5p mimics (main difference between mimics treatment and control treatment in WT Bcl-2 3′-UTR: *t*_4_ = 7.10, *P* = 0.0021). On the other hand, luciferase activity of mutant Bcl-2 3′-UTR was not affected by treatment with miR-34b-5p mimics (main difference between mimics treatment and control treatment in MUT Bcl-2 3′-UTR: *t*_4_ = 0.9156, *P* = 0.4117; Fig. 3d). These results indicated that Bcl-2 3′-UTR could be targeted by miR-34b-5p mimics which affected the stability or the translation efficiency of Bcl-2 mRNA. Then, to validate whether miR-34b-5p mediates apoptotic signaling by modulating Bcl-2, we evaluated the protein levels of Bcl-2, Bax, pro-caspase-3, and cleaved caspase-3 in primary astrocytes treated with miR-34b-5p mimics, miR-34b-5p inhibitors, and the respective control mimics or inhibitors. Astrocytes treated with vehicle and miR-34b-5p mimic controls or inhibitor controls have the same levels of Bcl-2, Bax, pro-caspase-3, and cleaved caspased-3. However, miR-34b-5p mimic treatment reduced the endogenous level of Bcl-2 but increased the Bax level (Fig. 4a). Conversely, miR-34b-5p inhibitors caused accumulation of Bcl-2 and pro-caspase-3 in astrocytes but reduction of Bax and cleaved caspase-3 (Fig. 4b). These data suggested that miR-34b-5p affected Bcl-2 and Bax protein levels and presented a pro-apoptotic function by modulating Bax/Bcl2 ratio expression (difference among five treatment groups, *F*_4,20_ = 123.8, *P* < 0.0001; Fig. 4c).

Finally, to confirm that the pro-apoptotic function of miR-34b-5p depends on Bcl-2, we performed co-transfection of Bcl-2 siRNA and miR-34b-5p inhibitors in primary astrocytes. Transfection of miR-34b-5p mimics and inhibitors affected miR-34b-5p level correspondently (Additional file 2: Fig. 2B, Fig. 3b). Reducing Bcl-2 alone using siRNA could induce cell apoptosis represented by upregulating cleaved caspase-3 levels. Conversely, miR-34b-5p inhibitor treatment alone inhibited cell death by reducing pro-caspase cleavage. However, astrocytes with miR-34b-5p treatment in the context of Bcl-2 siRNA silencing increased active caspase-3 level compared with the group treated with miR-34b-5p inhibitors alone, proving that miR-34b-5p induced cell apoptosis was Bcl-2 dependent (Fig. 4d–f). Interestingly, the Bcl-2 mRNA level remains stable in response to treatment with miR-34b-5p mimics or inhibitors, but the effects of miR-34b-5p on suppressing Bcl-2 mRNA translation efficiency have been proved by luciferase activity experiments and western blots (Figs. 3d, f, 4a, b). Thus, we think that the Bcl-2 protein suppression caused by miR-34b-5p is not achieved through affecting mRNA stability of Bcl-2 but that it could be achieved through translational repression mechanisms. Taken together, these results show that miR-34b-5p functions as a pro-apoptotic factor in astrocytes by mediating Bcl-2 protein levels.

## Discussion

Understanding neuronal apoptotic mechanisms is pivotal in developing novel therapies for neurodegenerative diseases and related CNS diseases. Neuronal apoptosis, closely linked with Alzheimer’s disease, Parkinson’s disease, and epilepsy, is triggered by osmolytic stress and DNA damage induced by accumulation of free radicals [6]. Specifically, neuronal death caused by seizure occurs partially from excessive Na^+^ and Ca^2+^ entry due to excitotoxic glutamatergic neurotransmission gating. Although certain processes, including death receptor activation, TNF/caspase-8 signaling, and Bax activation induced by release of intrinsic cytochrome complex, are identified as causes of neuronal cell death, detailed regulation steps and the molecular players involved still remain elusive to researchers [36]. In this paper, we utilised a flurothyl-induced recurrent convulsions model, explored whether microRNAs control astrocyte death, and discovered miR-34b-5p connected to astrocyte apoptosis by affecting Bcl-2 mRNA translation.

In the present study, we investigated hippocampal microRNA transcriptome in rats with recurrent convulsions and identified transcriptional alterations of multiple microRNAs. Besides the miR-34s family, some of our top hits, such as miR-204, miR-488, and miR-183, have been investigated and proposed to regulate apoptosis, inflammation, and ER stress in various systems [37–39]. Here, we focused primarily on miR-34b-5p. We observed that induction of miR-34b-5p began at 2 h after the recurrent convulsions model was established, remaining high till 1 day after the establishment of recurrent seizure model, then returning to normal after 3 days. This expression patent of miR-34b-5p delineates its involvement in modulating neuronal damage in the early stage of convulsion-caused damage. Given that preventing neuronal death in early disease stages would greatly improve therapeutic outcomes, therapies targeting miR-34b-5p may be beneficial [40].

Accumulated evidence now supports the essential role of microRNAs during the development of CNS diseases, especially convulsions and convulsion-related epilepsy. Array-based microRNA profiling in both an experimental convulsion model and human epilepsy detected expression changes as well as complex regulations for various microRNAs [41–43]. Though the majority of cell death we observed involved astrocytes, a small population of hippocampus neurons also underwent cell death in our model, signifying the potential role of miR-34b-5p in neuron death as well. Much as with our findings, a recent study by Sano et al. [23] found that upregulation of microRNA-34a and neuronal death co-existed and correlated with each other in a mouse seizure model. In our study, we explored the mechanisms of association of miR-34b-5p level with cell death in recurrent-seizure hippocampus. We found that miR-34b-5p was upregulated in astrocytes treated with kainic acid and discovered that the pro-apoptotic function of miR-34b-5p was Bcl-2 dependent. Interestingly, our results revealed that miR-34b-5p regulated Bcl-2 protein without interfering with its mRNA stability in a certain time window, indicating that miR-34b-5p represses translation of Bcl-2 mRNA before moving it towards RISC-dependent degradation. Indeed, several recent studies have demonstrated that a large number of microRNAs primarily initiate translational repression in their target before degrading their mRNA targets rather than cause mRNA degradation immediately [44–46].

## Conclusion

To summarise, the present study contributes to an emerging focus on the function of microRNAs in the brain. We discovered that miR-34b-5p is induced by recurrent seizures in a rodent model of recurrent convulsions and illustrated how miR-34b-5p mediates Bcl-2 dependent caspase-3 activation and cell apoptosis. Our findings demonstrate a crucial role for miR-34b-5p in seizure-induced brain damage and the possibility that miR-34b-5p could serve as a novel therapeutic target for treating various neurological diseases.

## Additional files

10.1186/s12868-016-0291-6 Representative images of TUNEL staining after astrocytes were treated with different concentrations of kainic acid; scale bar 50 μm. The experiment was repeated 5 times.
10.1186/s12868-016-0291-6 **A** Astrocytes was transfected with WT Bcl-2 3′-UTR or Mutant Bcl2 3′-UTR followed by treating either with control or miR-34b-5p mimics. miR-34b-5p level after transfection was shown to validate the transfection efficiency. **B** Electrophoresis picture shows the miR-34b-5p level in respond to different treatment, demonstrating the transfection of all the plasmids.

## Abbreviations

CNS: central nervous system
HCC: hepatocellular carcinoma
KA: kainic acid
qPCR: quantitative real time PCR
SE: status epilepsy
TUNEL: in situ terminal deoxynucleotidyltransferase mediated dUTP nick end labeling of fragmented DNA
miR-34b-5p m NC: miR-34b-5p mimics negative control
miR-34b-5p i NC: miR-34b-5p inhibitors negative control
