# Supplementary figures and images for: MicroRNA-34b mediates hippocampal astrocyte apoptosis in a rat model of recurrent seizures
Source: BMC Neurosci. 2016 Aug 11;17:56. doi: 10.1186/s12868-016-0291-6 (PMC4981991; doi:10.1186/s12868-016-0291-6)

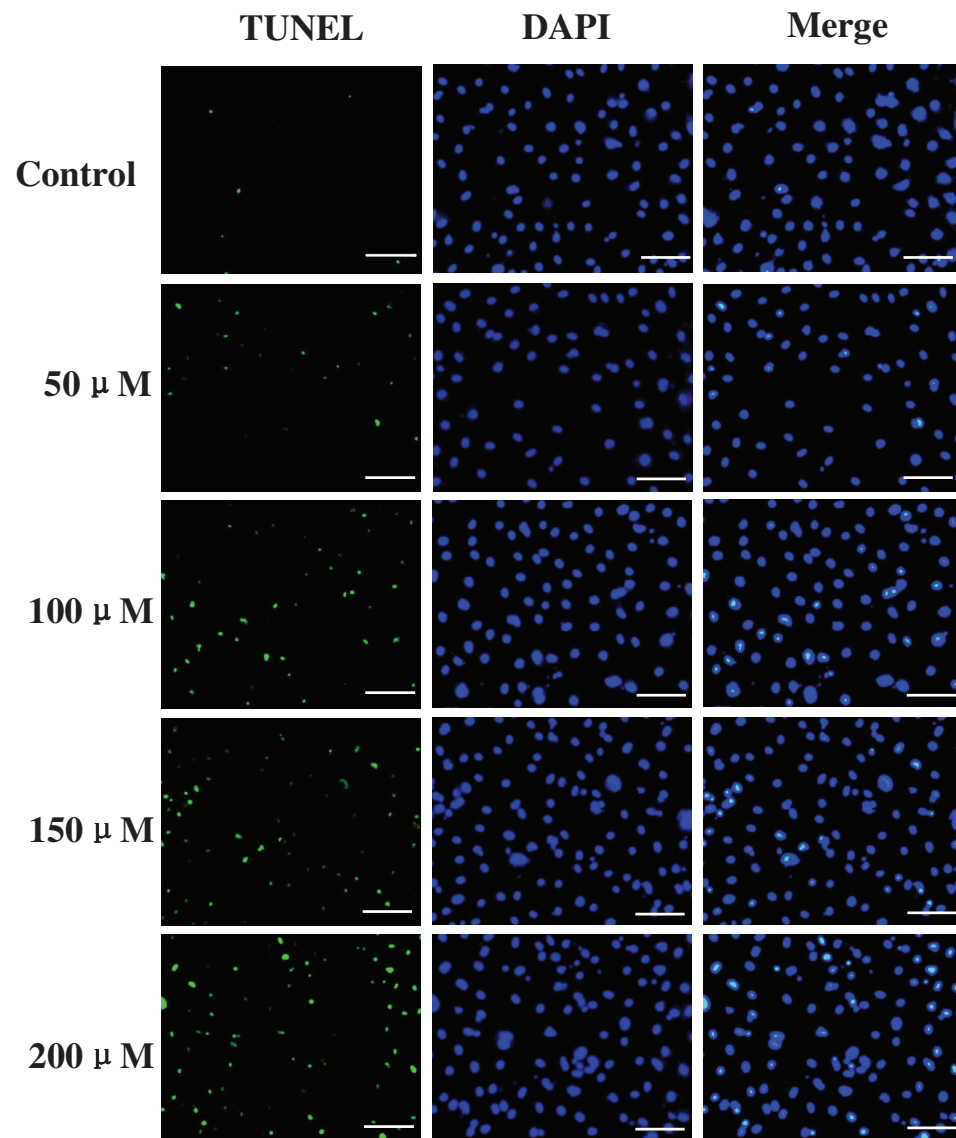

Supplement: Supplementary file 1 — 10.1186/s12868-016-0291-6 Representative images of TUNEL staining after astrocytes were treated with different concentrations of kainic acid; scale bar 50 μm. The experiment was repeated 5 times. [file 12868_2016_291_MOESM1_ESM.pdf]

A.

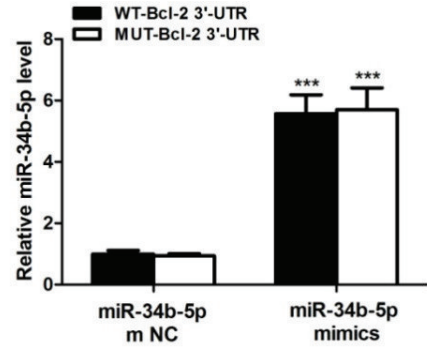

B.

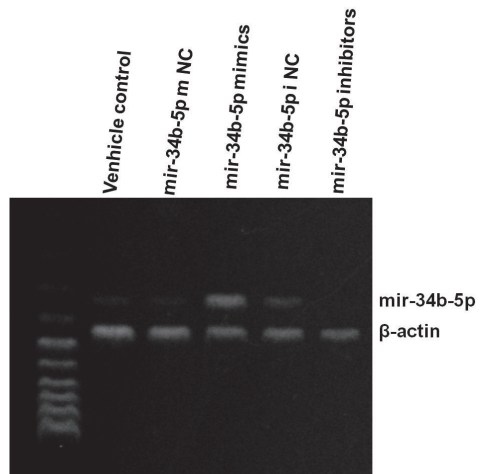

Supplement: Supplementary file 2 — 10.1186/s12868-016-0291-6 A Astrocytes was transfected with WT Bcl-2 3′-UTR or Mutant Bcl2 3′-UTR followed by treating either with control or miR-34b-5p mimics. miR-34b-5p level after transfection was shown to validate the transfection efficiency. B Electrophoresis picture shows the miR-34b-5p level in respond to different treatment, demonstrating the transfection of all the plasmids. [file 12868_2016_291_MOESM2_ESM.pdf]
